# Differences in perceptions of family functioning among adolescents with internalizing and externalizing disorders

**DOI:** 10.4103/0019-5545.74324

**Published:** 2010

**Authors:** Harpreet Mehar, T. M. Ismail Shihabuddeen

**Affiliations:** Department of Physical and Medical Rehabilitation PGIMER, - Dr. RML Hospital, New-Delhi-11000, India. E-mail: harpreetmehar@gmail.com; 1 Department of Psychiatry, Yenepoya Medical College and Hospital, Yenepoya University, Deralakatte, Mangalore - 575 018, India

Sir,

The incidence of several problem behaviors among adolescents is on the rise. This is of concern to us as we are aware that youngsters form the majority of population in India (56%) and they also have a very important role to play in the future of the country. So, it becomes important for us as mental health professionals to look into the matter. Among all the various etiological factors that play a role in contributing to deviant behavior, we do know the importance of the role that the family environment can play in the development of deviant behavior. Deviant behavior can include externalized and internalized problem behaviors.[1] We were interested in finding out any discrepancies in the family functioning of adolescents with behavioral problems of externalizing and internalizing disorders, using the Family Assessment Device (FAD)[2] on 15 families in each of the groups for internalizing and externalizing disorders. The results revealed that the adolescents of the two clinical groups differed significantly on the general functioning, problem solving, communication, and roles subscales of FAD as assessed by adolescents, and on general functioning, communication, roles, affective involvement, affective responsiveness, and problem-solving as assessed by the parents of adolescents with internalizing and externalizing disorders. The present research however suggests the specific domains of family functioning on which internalizing and externalizing disorders differ, and reveal parents having a higher discrepancy on domains of family functioning than adolescents of internalizing and externalizing disorders.[3] Found that negative parenting and exposure to deviant peers influenced aggression among adolescents. Peer groups in the present study were not studied. However the data are relevant in understanding the etiology of adolescents emotional and behavioural disorders and their management.
